# Optimal control strategies supported by system dynamics modelling: a study on hookworm disease in China

**DOI:** 10.1186/s40249-025-01293-w

**Published:** 2025-03-19

**Authors:** Huihui Zhu, Jinxin Zheng, Jilei Huang, Mizhen Zhang, Changhai Zhou, Tingjun Zhu, Hongchun Tian, Xiaohong Wu, Yang Liu, Bo Zhong, Hong Xie, Liping Zhang, Lei Tie, Jingwen Luo, Xiaoqin Mao, Bin Zhang, Xiu Deng, Suping Zhang, Menbao Qian, Shizhu Li, Xiaonong Zhou

**Affiliations:** 1 National Institute of Parasitic Diseases, Chinese Center for Disease Control and Prevention; Chinese Center for Tropical Diseases Research; National Key Laboratory of Intelligent Tracking and Forecasting for Infectious Diseases; Key Laboratory on Parasite and Vector Biology, Ministry of Health; WHO Centre for Tropical Diseases; National Center for International Research on Tropical Diseases, Ministry of Science and Technology, Shanghai, 200025 China; 2 School of Global Health, Chinese Center for Tropical Diseases Research, Shanghai Jiao Tong University School of Medicine, Shanghai, 200025 China; 3 Sichuan Center for Disease Control and Prevention, Chendu, China; 4 Hejiang Center for Disease Control and Prevention, Hejiang, China; 5 Luxian Center for Disease Control and Prevention, Luxian, China

**Keywords:** Hookworm disease, System dynamics, Intervention strategy, China

## Abstract

**Background:**

Hookworm disease remains a global health issue. In China, it persists with a 0.67% infection rate and uneven distribution in 2021. Optimized control strategies are needed. This study aims to optimize intervention strategies for hookworm disease in China.

**Methods:**

Structural analysis and parameter estimation were conducted using system dynamics theory. Key variables were identified via the Delphi method, leading to the creation of a causal loop diagram (CLD) and stock flow chart (SFC). Based on the SFC, parameter estimation and quantitative relationships were established and the model was validated. A cost-effectiveness model was then integrated into the intervention mechanism model. Various intervention measures were tested in the model to determine their cost-effectiveness ratio (CER) and effectiveness. Generalized linear models were constructed from simulation data, accounting for the impact of survey sites. The results were used to develop an optimized strategy for hookworm disease control.

**Results:**

In comparing drug treatment methods, whole population deworming (WPD) and key population deworming (KPD) showed lower CERs than examination and voluntarily deworming (EVD), saving 384.79–504.64 CNY and 354.35–506.21 CNY per infection reduced, respectively (*P* < 0.001). For WPD or KPD alone, CER decreased with increased drug coverage. For examination and deworming (ED) and EVD, CER was highest at 30% coverage for a 1-year intervention, but at 90% coverage for 2–5 years (*P* < 0.05). WPD, ED, and EVD had higher infection reduction rates than KPD, with ratios of 0.14–0.25, 0.10–0.19, and 0.08–0.17, respectively, over 1–5 years (*P* < 0.001). Continuous health education over 1–5 years showed that increasing coverage from a 10% baseline led to enhancing cost-effectiveness and intervention outcomes.

**Conclusions:**

In high-endemic areas (infection rate ≥ 20%) in China, prioritize WPD for better cost-effectiveness and outcomes. In medium-endemic areas (5% ≤ infection rate < 20%) where WPD isn't feasible, use ED for cost-effectiveness and KPD for infection reduction, based on local needs. In low-endemic areas (infection rate < 5%), encourage voluntary examination and treatment due to limited cost-effectiveness of mass treatment. Combining drug treatment with extensive health education can enhance long-term control effect. This strategy can guide control efforts for hookworm diseases in China.

*Clinical trial number*: Not applicable.

**Graphical Abstract:**

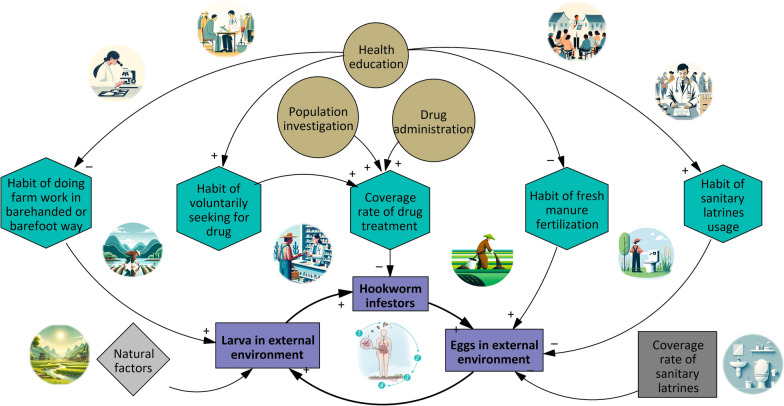

**Supplementary Information:**

The online version contains supplementary material available at 10.1186/s40249-025-01293-w.

## Background

Hookworm, classified as a soil-transmitted helminth (STH), encompasses several species, such as *Ancylostoma duodenale, Necator americanus, A. ceylanicum* and *A. caninum.* However, the primary culprits responsible for human hookworm diseases are *A. duodenale* and *N. americanus* [1, 2]*,* which primarily induce iron deficiency anemia and malnutrition [3–6], leading to lethargy, impaired physical and cognitive development for children and unfavorable pregnancy outcomes for women [2, 7]. Hookworm disease exhibits a significant prevalence worldwide, especially in sub-Saharan Africa, East Asia, the Pacific Islands, South Asia, Latin America, and the Caribbean [2, 5, 7, 8]. The global burden of hookworm infection was staggering, causing an estimated loss of 2.2 million disability-adjusted life-years in 2013 [8]. In the People’s Republic of China, the national survey in 2015 indicated an infection rate of 2.62% for hookworm, corresponding to an estimated 16.97 million infected individuals [9]. Moreover, data from national surveillance conducted between 2016 and 2021 revealed a declining trend in hookworm infection rates, with infection rates of 1.34%, 1.00%, 0.89%, 0.84%, 0.51%, and 0.67%, respectively. Despite the overall low prevalence, hookworm disease is widespread with regional disparities in China. Moreover, hookworm remains the dominant STH species other than *Ascaris lumbricoides* and *Trichuris trichuria* [10–15].

Hookworm disease has been classified as a neglected tropical disease (NTD) that required enhanced control efforts by the World Health Organization (WHO). It was also recognized as one of the infectious diseases that need to be eliminated in pre-SAC and SAC addressed in the 2030 Sustainable Development Goals (SDGs) set forth by the United Nations [7, 16, 17]. In the document of “Ending the neglect to attain the Sustainable Development Goals: the road map for neglected tropical diseases 2021–2030” issued by WHO in 2020, the 2030 targets for soil-transmitted helminthiases (STHs) control programs were also tackled, clarifying the targets and indicators to be achieved by 2030 [18, 19]. In China, the criteria for transmission control and interruption of STHs was issued in 2018, which elucidated the criteria for transmission control and interruption of STHs in county level as: the number of people surveyed exceeds 1000 each year, and the infection rate of soil-transmitted helminths has remained below 1%/0.1% (1% for transmission control and 0.1% for transmission interruption) for 3 consecutive years; Meanwhile, there is at least one person proficient in soil-transmitted helminth microscopy examination techniques. Moreover, comprehensive records and documentation of soil-transmitted helminthiasis prevention and control efforts are maintained [20]. The criteria offered an instruction to promote the control and elimination of STHs.

Given current epidemiological status of hookworm disease in China and the urgent to advance efforts towards the control and eventual elimination of hookworm disease in accordance to the 2030’s targets of WHO, there is a pressing need for a quantitative, cost-effective, and context-specific strategy for the control of hookworm disease. Studies have identified factors influencing hookworm disease, such as temperature, gender, economic status, and hygiene practices like handwashing and working with barefoot [21–25]. Other studies have also confirmed the effectiveness of interventions, including drug administration and water, sanitation, and hygiene (WASH) improvements, in controlling the disease [23, 26–38]. However, there has been a gap in systematic research conducted to assess the impact of multiple intervention measures as a cohesive system. This includes evaluating the individual effects of each intervention measure, as well as the potential changes in intervention levels, on the control of hookworm disease. Specifically, it is essential to explore the role of various intervention measures and determine their optimal combination in preventing the transmission of hookworm disease.

System dynamics, an interdisciplinary field derived from system theory, offers a promising approach to addressing these challenges. System theory is a theory that uses logical and mathematical methods to study system behaviors by analyzing interrelationships and dynamics among components. Originating from systems theory, system dynamics could synthesize data from multiple sources and integrate various types of methodologies for research [39]. Established in 1956, system dynamics has rapidly gained widespread application and global recognition in less than 70 years [39, 40]. Initially utilized in industries, management control, finance, and other fields, system dynamics has now found its place in the realm of public health [41–51], and the application in public health is increasing [52–61], yet there remains a scarcity of research on the use of system dynamics model for the control of parasitic diseases [41, 62]. This study represents the first attempt to apply system dynamics model in the context of hookworm disease control.

This study aims to develop a model for intervening in transmission of hookworm disease using system dynamics modeling, and to analyze the effect and cost-effectiveness of various control methods in order to derive optimized strategies for tackling hookworm disease in China.

## Methods

The entire process of this study included model assumption, model development, model validation, and model simulation. The schematic representation of the research project could be found in Supplementary Figure S1.

### Model assumption

In system dynamics, there is no ultimate model; a model is a relative achievement that meets specific predetermined requirements. Modeling the same system from different perspectives can yield multiple views, each describing only one aspect of the actual system [39]. Therefore, it is crucial to determine which system aspects need description according to the modeling purpose. Assumptions were developed to ensure model rigor and clarify applicability: (i) the model specifically targeted hookworm disease caused by *N. americanus*: *N. americanus* was the primary endemic species in China, which differed from *A. duodenale* in transmission and biological features [63–65]. This target aimed to develop an effective intervention mechanism tailored to current control needs; (ii) exclusion of mortality: Given the low infection intensity and minimal fatalities from hookworm in China, mortality was excluded from the model to streamline its structure and concentrate on primary concerns; (iii) no reinfection during initial infection period: To avoid structural complexity, the model assumed no reinfection occurs during the initial infection period, but reinfection was possible after recovery.

### Model development

**Fig. 1:**
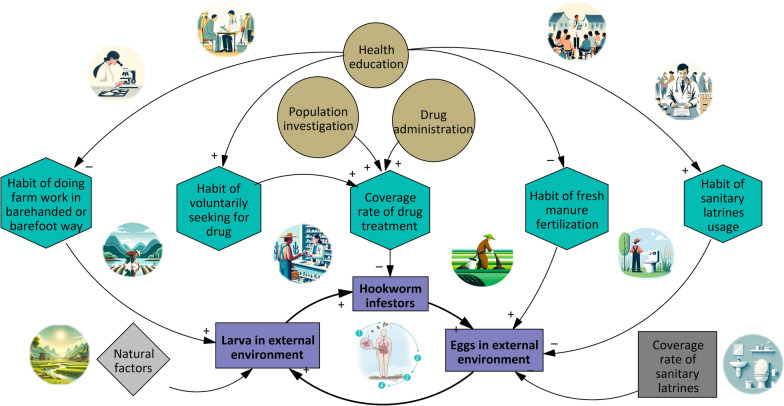
Causal loop diagram (CLD) of the interventional mechanism model of hookworm disease

**Fig. 2:**
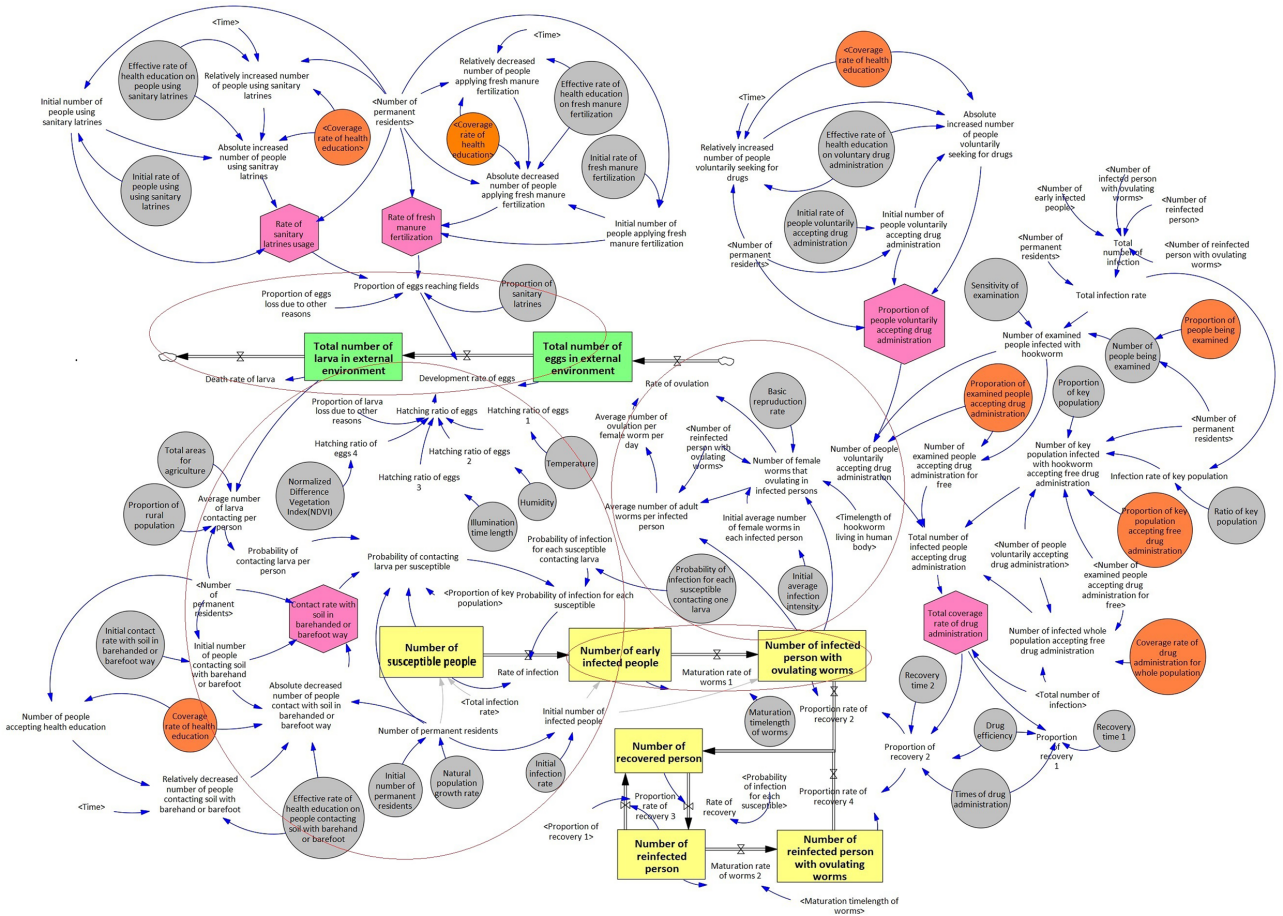
Stock flow chart (SFC) of the interventional mechanism model of hookworm disease

Model development included these following steps:

1. System analysis: To analyze the internal feedback structure and dynamic behavior within the hookworm disease transmission process based on system dynamics theory [39, 40]. By doing this, the system boundaries and the interrelationships among major system modules were clarified and presented in System Framework Diagram (Supplementary Figure S2).
2. Indicator system confirmation by Delphi method [66–68]: Indicators were gathered through a literature review based on the System Framework Diagram. Ten experts with rich experience in parasitic disease control were then convened to refine and expand the indicators through brainstorming, creating a primary indicator system. A consultation questionnaire was designed from this system. Experts were selected based on: (i) Over 5 years of experience in parasitic disease prevention or research; (ii) Experience in research or fieldwork on parasitic diseases; (iii) Interest in the study and willingness to participate. Questionnaire results were analyzed using Excel 2010 (Microsoft, Redmond, United States). Indicators deemed important by at least 70% of the experts were included to establish the final indicator system.
3. Structural analysis: The Vensim® DSS 9.3.5 Software (Ventura Systems, Inc., United States, herein after referred to as “Vensim software”) was utilized for model development. Structural analysis was performed based on system analysis and the validated indicator system, with the results presented in the CLD (Fig. 1). Quantity variables were defined in accordance with the selected indicators and the CLD, and the causal relationships between different variables were illustrated in the stock flow chart (SFC) [39, 40] (Fig. 2).
4. Parameter estimation and determination of quantity relationships were conducted based on the SFC. The relationships between state variables and rate variables were confirmed using integral functions within the Vensim Software, with the following formula [39]:

$${\text{Stock}}\left( t \right) = \int^{{\text{t}}}_{{{\text{t}}0}} \left[ {{\text{Inflow}}\left( s \right) - {\text{ Outflow}}\left( s \right)} \right]{\text{d}}s + {\text{ Stock}}\left( {t_{0} } \right)$$

Among these variables, Stock (*t*) denoted the stock quantity at time t, Inflow (*s*) represented the inflow quantity, and Outflow (*s*) represented the outflow quantity. Stock (t_0_) refered to the initial stock quantity at time t_0_.

Flow was a rate variable, defined as the derivative of Stock, and can be mathematically expressed by a differential formula:

$${\text{d}}\left( {{\text{Stock}}} \right)/{\text{d}}t = {\text{Inflow}}\left( t \right) - {\text{Outflow}}\left( t \right)$$

Time (*t*) was a continuous variable in the model, which was utilized to depict the evolving trends of various variables over time.

Other than these, constants and exogenous variables were gathered through literature sources, statistical yearbooks, authoritative websites, and field investigations. The quantitative relationships among auxiliary variables were validated using intuitive quantitative descriptions, machine learning, distribution simulation, lookup functions, and other mathematical or logical functions within the Vensim Software. The details were listed as follows:

1. Intuitive quantitative relationship description: There were intuitive quantitative relationships among some variables in light of professional knowledge. For instance, the infection rate was the number of infected individuals divided by the total population, and the total population was the sum of infected and non-infected individuals. Additionally, the rate of hygiene knowledge and hygiene behaviors were all descripted intuitively.
2. Field investigation: In model simulation, certain initial values reflecting local conditions, infection status, and prevention measures were sourced from field investigations. These included the initial hookworm infection rate, initial health education coverage rate, initial rate of contacting soil with barefoot, initial proportion of individuals voluntarily taking medication, initial rate of fresh manure fertilization, initial usage rate of sanitary latrines, etc.
3. Obtaining from literature, statistical yearbooks, and authoritative websites: Key parameters related to hookworm, such as the basic reproduction rate, the duration for hookworm eggs to develop into larvae, the survival duration of larvae in the external environment, and the time length from larvae invasion to adult development in humans, together with the quantitative relationships between certain variables, such as the relationship between the average daily egg production of female hookworms and the number of adult worms in the body were all obtained from literature [63–65]. Local data, such as local area, total population, and rural population ratio were sourced from local statistical yearbooks. Moreover, historical local data on temperature, humidity, vegetation cover index, and sunlight duration were obtained from the Resource and Environment Science and Data Center of the Chinese Academy of Sciences (https://www.resdc.cn/data.aspx?DATAID=349). Monthly observational datasets from meteorological stations in China were converted into raster data using ArcGIS 10.2 software (Esri, Redlands, United States) and interpolated to extract local data to serve as exogenous variables in the model.
4. Artificial intelligence algorithms: Machine learning was employed to analyze the main factors influencing hookworm disease and the dependencies between these factors and the disease. These included examining the quantitative relationships between temperature, humidity, rainfall, normalized vegetation cover index, sunlight duration, and hookworm infection rates.
5. Distribution simulation: The probability of larvae contacting with the human body, as well as the probability of successful infection after contact were described based on the principle of the Poisson distribution, thereby characterizing the entire process of larvae infecting the human body during the infection period [69].
6. Lookup function [39]: In modeling by Vensim Software, nonlinear relationships between certain variables that were difficult to achieve through simple simulations, yet could be accomplished using lookup functions. The steps to establish a lookup function were as follows: (1) Identifying the independent and dependent variables; (2) Determining the variables' value ranges using historical and forecast data; (3) Considering the curve's shape and slope to reflect the relationship between variables; (4) Choosing appropriate endpoints and inflection points; (5) Creating a graph with the X-axis for the independent variable and the Y-axis for the dependent variable.

The mathematical description of a lookup function in Vensim was as follows:

$${\text{Lookup name}}\left( {\left[ {\left( {{\text{Xmin}} - {\text{Xmax}}} \right) \, - \, \left( {{\text{Ymin}},{\text{ Ymax}}} \right)} \right], \, \left( {{\text{X1}},{\text{ Y1}}} \right), \, \left( {{\text{X2}},{\text{ Y2}}} \right), \, ..., \, \left( {{\text{Xn}},{\text{ Yn}}} \right)} \right)$$

In this study, the relationships between temperature, humidity, daylight duration, the normalized difference vegetation index (NDVI) and the hookworm larval hatching rate were described using lookup functions. This was done by referring to the dependencies between these factors and hookworm infection rates obtained from machine learning.

(7) Other functions [39]: Vensim software offers various mathematical, logical and delay functions to facilitate modeling, beyond basic arithmetic functions. Examples include:
  EXP(X) = e^x^, the exponential function, where e = 2.718…;
  INTEGER(X), which converts X to an integer.

In this study, the INTEGER function was used for variables referring to number of eggs or people, such as the number of female worms ovulating eggs, and the reduction in people using fresh manure as fertilizer. The EXP function was used to describe the average daily egg production per female worm, the probability of hookworm contact, and the infection probability for each susceptible individual; Logical functions of IF THEN ELSE was used to return different values according to the situations defined. In this study, the IF THEN ELSE function was applied to almost all rate variables, ensuring no outflow when state variable values were zero; It takes approximately 70 days from invading the human body to maturing into adult hookworms, and this process was depicted in the model using the DELAY1 function.

The model was primarily developed with the finish of SFC, the completion of parameter estimation and determination of quantity relationships.

### Model validation

Structural validation [40] ensures the rationality of a model's structure and behavior, encompassing direct structural validation and behavior validation specific to the structure.

1. Direct structural validation: This involved evaluating the model's structure, boundaries, and dimensional consistency. The boundary adequacy test ensured all relevant aspects are included using a boundary checklist to identify missing key variables; Structural assessment checked if the model's structure was logical and functional, with all variables in feedback loops, initial values set, and feedback relationships defined. This was done by clicking the “Check model” button in Vensim software, where a successful check results in a “Model is OK” message. Errors were indicated otherwise; Dimensional Consistency test ensured mathematical equations had consistent dimensions, verified by a “Units Check” button in Vensim software, resulting in a “Units are Ok” message if passed.
2. Behavior validation specific to the structure: Verified if the model's logic reflected real scenarios and if parameters were reasonable. Integration error testing assessed sensitivity to time step choices by comparing model results with different time steps. Significant changes necessitated a smaller time step; Sensitivity testing evaluated if model outcomes aligned with the actual system when input values change, ensuring reasonable changes in other variables.

### Field validation

Sichuan province ranked the second highest hookworm infection rates in China according to national surveillance in 2018 [10]. Previous investigation estimated prevalence rates of about 10% in Hejiang County and 20% in Lu County, so these two counties were selected to represent two prevalence levels for this study. Additionally, towns with similar hookworm prevalence and socio-environmental conditions were chosen as intervention and control sites within these counties respectively.

Baseline survey, field intervention, and post-intervention survey were conducted in 2019, 2019–2020, and 2021, respectively. The field data from the baseline survey in 2019 served as the starting point, and the data from the intervention survey in 2021 served as the endpoint for simulation, with the time step setting as 1 day.

Drawing from field data on the natural environment, population size and composition, initial infection rate, and initial qualification rate of hygiene habits, intervention measures were implemented at each survey spot. These measures included conducting investigations, administering drugs, and providing health education to the population. The aim was to predict the post-intervention qualification rate of hygiene habits and infection rates at the survey spots. Comparing the simulation results to the field survey data allowed for the evaluation of the model's accuracy, with the relative error being calculated.

$${\text{Relative error}}\, = \left| {{\text{Real value}} - {\text{Prediction value}}} \right|/{\text{Real value}}$$

### Model simulation

Following the validation of the model, a cost-effectiveness model was developed based on the intervention mechanism model and utilizing field data obtained from survey spots.

In the simulation of drug administration, four different drug administration measures were systematically evaluated at varying levels ranging from 0.1 to 0.9, with the step of 0.1. The CER for reducing per number of infected individuals and per corresponding proportions of infection rate decrease were calculated for each measure. Subsequently, a dataset was generated from the simulation results, and generalized linear models were constructed incorporating the aforementioned variables using R4.3.1 software (R Core Team). The initial infection rate served as the dependent variable, while the CER and proportion of infection rate decrease were included as independent variables. Additionally, adjustments were made to account for the impact of survey sites on the model outcomes.

In the simulation of health education, varying levels of coverage rates for health education were introduced into the model, and CER for increasing per qualified individuals of hygiene behaviors was obtained. Generalized linear models were similarly developed, incorporating the aforementioned variables, with the initial qualified rate of hygiene behaviors serving as the dependent variable and the CER and proportion for increasing per qualified individuals of hygiene behaviors as independent variables. In addition, adjustments were made to account for the impact of survey sites on the model outcomes. Through this analysis, the CER and effects of various control measures were determined, enabling the identification of optimal control strategies under different conditions through a comprehensive evaluation.

## Results

### Model development

System analysis revealed that the key factors influencing hookworm disease include natural, social, and biological factors, as well as the intervention measures implemented during prevention and control efforts. These factors are incorporated within the system boundary, and their interrelationships are depicted in the System framework diagram (Supplementary Figure S2).

Based on this diagram, an indicator system consisting of 33 indicators across 5 major categories was established. A total of 40 questionnaires were distributed, with a 100% response rate. Expert opinions (Supplementary Figure S3) were consulted to identify key indicators, leading to the construction of a CLD that illustrates the impact of intervention measures on hookworm transmission through key interface variables (Fig. 1).

The SFC illustrating the causal relationships among variables was developed (Fig. 2). The variables within circles represent the initial variables. Among them, the variables in gray circles depict the actual conditions of the survey spots, typically derived from field surveys, yearbooks, or literature data. The variables in orange circles represent the intervention measures applied. The variables within rectangular boxes are cumulative variables, where yellow indicates the cumulative status of infected individuals within the population, and green represents the accumulation of eggs/larvae in the external environment. The variables within pink hexagonal boxes are key intermediate variables in the model, which are generally targeted by intervention measures to influence the prevalence of hookworm in survey spots.

The SFC illustrates the transmission process of hookworm, along with the impact of influencing factors and intervention measures on hookworm prevalence. The inner circle of the model depicts the life cycle of the hookworm, divided into four sections corresponding to the four red circles/ovals in the SFC. Starting from the far right and moving counterclockwise, these sections are: (i) The excretion of eggs by infected individuals into the external environment; (ii) The development of eggs into infective larvae in the external environment; (iii) The infection of humans by the infective larvae; and (iv) The maturation of larvae into adult worms within the human body, followed by egg production. The outermost part of the SFC illustrates the influence of external intervention factors on the transmission process of hookworm disease through key intermediate variables. The names and expressions of variables are detailed in Supplementary Tables S1–S4.

### Model validation

#### Structural validation

The boundary adequacy assessment confirmed that all key variables relevant to the model's objectives are included, as detailed in Tables S1–S4. Using Vensim software, the model passed structural and dimensional consistency checks, receiving “Model is OK” and “Units are OK” notifications.

Dimensional consistency checks are to verify the adequacy of a 1-day time step, simulations were conducted using baseline data from Fengming Town, Hejiang County, with time steps of 1, 0.5, 0.25, 0.125, 0.0625, and 0.03125 days respectively. As shown in Figure S4, the simulation outcomes for key variables were nearly identical across these time steps, confirming that a 1-day time step is sufficient.

Using the same baseline data, the model's sensitivity was assessed across three scenarios: variations in the contact rate with soil in barehanded or barefoot way, simultaneous variations in the compliance rates of four hygiene behaviors (contact rate with soil in barehanded or barefoot way, rate of sanitary latrines usage, rate of fresh manure fertilization, and coverage rate of drug treatment), and variations in proportion of people being examined. As shown in Figure S5, the model demonstrated sensitivity, with key variables like infection rate, the number of infected persons with ovulating worms, and number of recovered persons showing increased variation rates as input values changed, demonstrating model sensitivity.

#### Field validation

Data from the survey spots in Hejiang County and Lu County for the years 2019–2021 was simulated by incorporating field survey data into the model. The simulation results for key variables, including number of early infected persons, number of infected persons with ovulating worms, and infection rate, are presented in Figure S6. Fengming Town in Hejiang County and Baihe Town in Luxian County were designated as intervention groups, while the other two towns served as control groups.

The simulation results indicate that the number of early infected persons at all survey spots was initially high, gradually transitioning to persons with ovulating worms over time. Moreover, the infection rates of all spots showed declining trends in overall infection rates, with intervention groups experiencing a more rapid decrease due to intervention measures. Additionally, the relative errors of the qualification rate of hygiene habits and infection rate were calculated, and found to be within a 15% margin of relative error (Supplement Tables S5–S6).

###  Model simulation

**Fig. 3:**
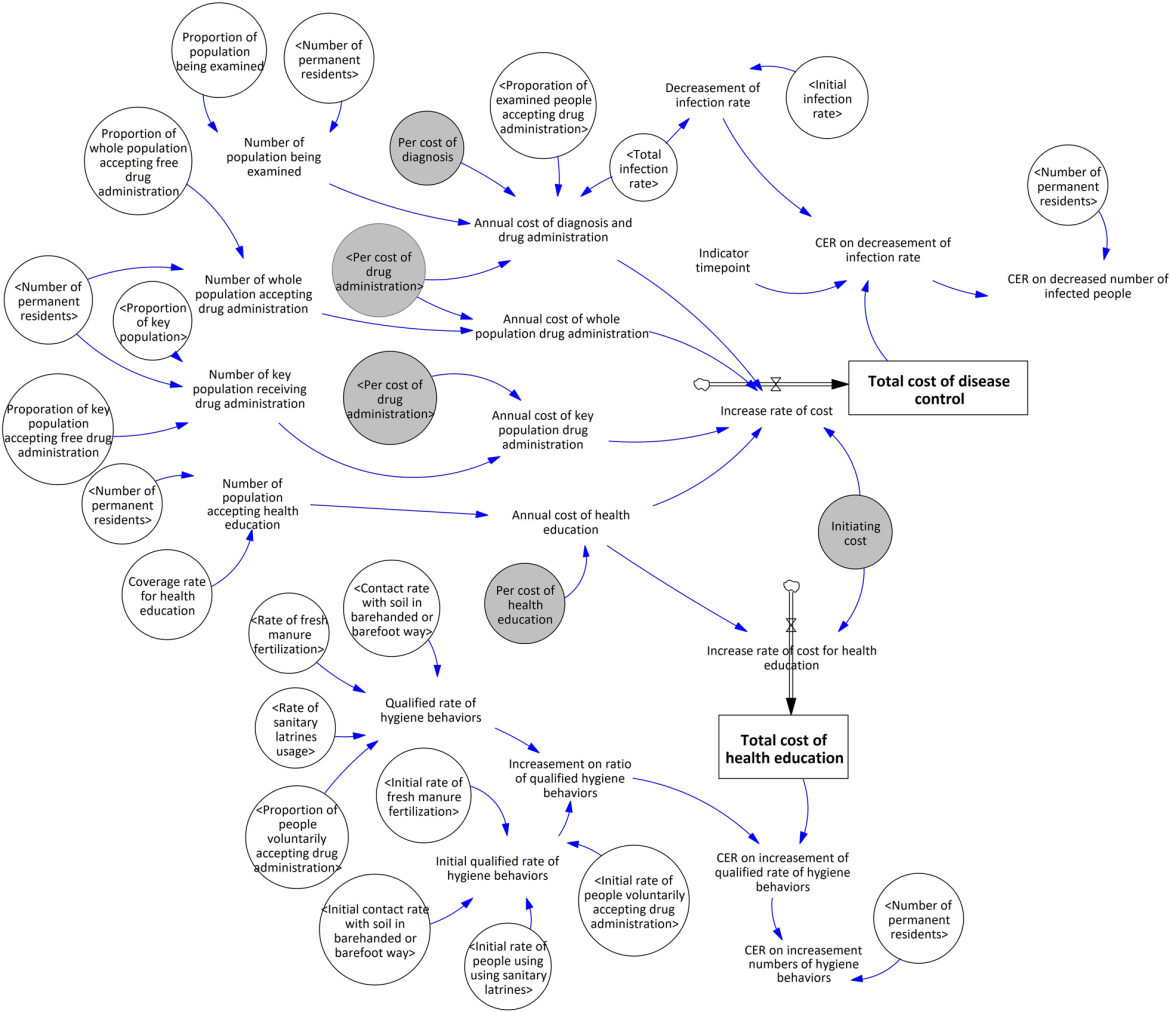
Stock flow chart (SFC) of the cost-effectiveness model of hookworm disease

The SFC and variable expressions of the cost-effectiveness model are depicted in Fig. 3 and Supplementary Table S7, respectively. Results of generalized linear models were presented in Tables 1, 2, 3.

**Table 1:** Analysis of the cost-effectiveness ratio of different drug administration methods on decrease of infected people by generalized linear models based on data from model simulation

Independent variables	1 year		2 years		3 years		4 years		5 years	
	Adjusted coefficient^b^	*P* value	Adjusted coefficient^b^	*P* value	Adjusted coefficient^b^	*P* value	Adjusted coefficient^b^	*P* value	Adjusted coefficient^b^	*P* value
MDA^a^										
EVD	1 (REF)	/	1 (REF)	/	1 (REF)	/	1 (REF)	/	1 (REF)	/
WPD	− 384.79	< 0.001	− 298.77	< 0.001	− 381.09	< 0.001	− 430.55	< 0.001	− 504.64	< 0.001
KPD	− 354.35	< 0.001	− 287.10	< 0.001	− 368.71	< 0.001	− 425.67	< 0.001	− 506.21	< 0.001
ED	− 35.47	> 0.05	− 32.11	> 0.05	− 30.95	> 0.05	− 24.97	> 0.05	− 23.58	> 0.05
CDA^a^										
10%	1 (REF)	/	1 (REF)	/	1 (REF)	/	1 (REF)	/	1 (REF)	/
20%	− 59.08	> 0.05	− 40.78	> 0.05	− 48.68	> 0.05	− 40.13	> 0.05	− 37.50	> 0.05
30%	− 72.82	< 0.05	− 46.33	> 0.05	− 51.55	> 0.05	− 35.09	> 0.05	− 25.74	> 0.05
40%	− 75.62	< 0.05	− 43.97	> 0.05	− 43.88	> 0.05	− 18.87	> 0.05	− 1.24	> 0.05
50%	− 74.01	< 0.05	− 38.42	> 0.05	− 31.74	> 0.05	2.51	> 0.05	29.45	> 0.05
60%	− 70.14	< 0.05	− 31.16	> 0.05	− 17.08	> 0.05	27.04	> 0.05	64.06	> 0.05
70%	− 64.92	< 0.05	− 22.82	> 0.05	− 0.69	> 0.05	53.85	> 0.05	101.52	> 0.05
80%	− 58.27	> 0.05	− 13.15	> 0.05	18.22	> 0.05	84.49	> 0.05	144.11	< 0.05
90%	− 52.54	> 0.05	− 4.56	> 0.05	34.47	> 0.05	110.40	< 0.05	179.72	< 0.01
MDA^a^										
WPD	1 (REF)	/	1 (REF)	/	1 (REF)	/	1 (REF)	/	1 (REF)	/
KPD	30.16	< 0.05	11.51	> 0.05	12.17	> 0.05	4.60	> 0.05	− 1.91	> 0.05
CDA^a^										
10%	1 (REF)	/	1 (REF)	/	1 (REF)	/	1 (REF)	/	1 (REF)	/
20%	− 76.93	< 0.01	− 49.04	< 0.001	− 63.83	< 0.01	− 62.74	< 0.01	− 66.35	< 0.05
30%	− 100.30	< 0.001	− 61.93	< 0.001	− 79.82	< 0.001	− 77.49	< 0.01	− 80.93	< 0.01
40%	− 110.98	< 0.001	− 67.04	< 0.001	− 85.54	< 0.001	− 81.63	< 0.01	− 83.90	< 0.01
50%	− 116.67	< 0.001	− 69.18	< 0.001	− 87.29	< 0.001	− 81.57	< 0.01	− 82.22	< 0.01
60%	− 119.87	< 0.001	− 69.84	< 0.001	− 87.05	< 0.001	− 79.35	< 0.01	− 78.12	< 0.01
70%	− 121.65	< 0.001	− 69.65	< 0.001	− 85.64	< 0.001	− 75.83	< 0.01	− 72.57	< 0.01
80%	− 122.53	< 0.001	− 68.91	< 0.001	− 83.46	< 0.001	− 71.46	< 0.01	− 66.06	< 0.05
90%	− 121.49	< 0.001	− 67.04	< 0.001	− 79.82	< 0.001	− 65.22	< 0.01	− 57.35	< 0.05
MDA^a^										
ED	1 (REF)	/	1 (REF)	/	1 (REF)	/	1 (REF)	/	1 (REF)	/
EVD	35.47	< 0.001	32.11	< 0.001	30.95	< 0.05	24.97	> 0.05	23.58	> 0.05
CDA^a^										
10%	1 (REF)	/	1 (REF)	/	1 (REF)	/	1 (REF)	/	1 (REF)	/
20%	− 41.23	< 0.05	− 32.52	> 0.05	− 33.52	> 0.05	− 17.52	> 0.05	− 8.65	> 0.05
30%	− 45.34	< 0.05	− 30.74	> 0.05	− 23.28	> 0.05	7.31	> 0.05	29.44	> 0.05
40%	− 40.25	< 0.05	− 20.91	> 0.05	− 2.22	> 0.05	43.89	> 0.05	81.41	> 0.05
50%	− 31.35	> 0.05	− 7.66	> 0.05	23.80	> 0.05	86.58	< 0.05	141.11	< 0.01
60%	− 20.41	> 0.05	7.52	> 0.05	52.90	0.05	133.43	< 0.001	206.24	< 0.001
70%	− 8.19	> 0.05	24.01	> 0.05	84.25	< 0.01	183.53	< 0.001	275.62	< 0.001
80%	5.98	> 0.05	42.62	< 0.05	119.90	< 0.001	240.43	< 0.001	354.28	< 0.001
90%	17.72	> 0.05	58.69	< 0.01	149.68	< 0.001	287.27	< 0.001	418.32	< 0.001

The “/” means that the independent variable was set as the reference (REF), therefore, there was no need to provide the *P* value

^a^*MDA* Methods of drug administration; *CDA* Coverage rate of drug administration

^b^Effects of different survey spots adjusted

**Table 2:** Analysis on the decrease ratio of infection rate for various drug administration methods by generalized linear models based on data from model simulation

Independent variables	1 year		2 years		3 years		4 years		5 years	
	Adjusted coefficient^b^	*P* value	Adjusted coefficient^b^	*P* value	Adjusted coefficient^b^	*P* value	Adjusted coefficient^b^	*P* value	Adjusted coefficient^b^	*P* value
MDA^a^										
KPD	1 (REF)	/	1 (REF)	/	1 (REF)	/	1 (REF)	/	1 (REF)	/
WPD	0.14	< 0.001	0.23	< 0.001	0.26	< 0.001	0.26	< 0.001	0.25	< 0.001
ED	0.10	< 0.001	0.16	< 0.001	0.19	< 0.001	0.20	< 0.001	0.19	< 0.001
EVD	0.08	< 0.001	0.13	< 0.001	0.16	< 0.001	0.17	< 0.001	0.17	< 0.001
CDA^a^										
10%	1 (REF)	/	1 (REF)	/	1 (REF)	/	1 (REF)	/	1 (REF)	/
20%	0.05	< 0.001	0.10	< 0.001	0.14	< 0.001	0.97	> 0.05	0.17	> 0.05
30%	0.10	< 0.001	0.19	< 0.001	0.25	< 0.001	2.23	> 0.05	0.31	> 0.05
40%	0.14	< 0.001	0.26	< 0.001	0.34	< 0.001	3.74	> 0.05	0.41	> 0.05
50%	0.18	< 0.001	0.33	< 0.001	0.42	< 0.001	5.59	> 0.05	0.49	> 0.05
60%	0.22	< 0.001	0.39	< 0.001	0.48	< 0.001	7.69	< 0.05	0.55	< 0.05
70%	0.25	< 0.001	0.44	< 0.001	0.53	< 0.001	10.14	< 0.01	0.59	< 0.01
80%	0.29	< 0.001	0.49	< 0.001	0.58	< 0.001	12.55	< 0.01	0.63	< 0.01
90%	0.31	< 0.001	0.52	< 0.001	0.61	< 0.001	14.60	< 0.01	0.66	< 0.01
MDA^a^										
WPD	1 (REF)	/	1 (REF)	/	1 (REF)	/	1 (REF)	/	1 (REF)	/
KPD	− 0.14	< 0.001	− 0.23	< 0.001	− 0.26	< 0.001	− 0.26	< 0.001	− 0.25	< 0.001
CDA^a^										
10%	1 (REF)	/	1 (REF)	/	1 (REF)	/	1 (REF)	/	1 (REF)	/
20%	0.05	< 0.05	0.10	< 0.001	0.13	< 0.001	0.17	< 0.001	0.19	< 0.001
30%	0.10	< 0.001	0.18	< 0.001	0.24	< 0.001	0.29	< 0.001	0.32	< 0.001
40%	0.14	< 0.001	0.25	< 0.001	0.33	< 0.001	0.39	< 0.001	0.42	< 0.001
50%	0.18	< 0.001	0.32	< 0.001	0.40	< 0.001	0.46	< 0.001	0.49	< 0.001
60%	0.21	< 0.001	0.37	< 0.001	0.46	< 0.001	0.52	< 0.001	0.54	< 0.001
70%	0.24	< 0.001	0.42	< 0.001	0.51	< 0.001	0.57	< 0.001	0.59	< 0.001
80%	0.28	< 0.001	0.46	< 0.001	0.55	< 0.001	0.60	< 0.001	0.62	< 0.001
90%	0.30	< 0.001	0.50	< 0.001	0.59	< 0.001	0.63	< 0.001	0.64	< 0.001
MDA^a^										
ED	1 (REF)	/	1 (REF)	/	1 (REF)	/	1 (REF)	/	1 (REF)	/
EVD	− 0.02	< 0.001	− 0.03	< 0.001	− 0.03	< 0.001	− 0.03	< 0.001	− 0.03	< 0.001
CDA^a^										
10%	1 (REF)	/	1 (REF)	/	1 (REF)	/	1 (REF)	/	1 (REF)	/
20%	0.05	< 0.001	0.10	< 0.001	0.14	< 0.001	0.18	< 0.001	0.21	< 0.001
30%	0.10	< 0.001	0.19	< 0.001	0.26	< 0.001	0.32	< 0.001	0.35	< 0.001
40%	0.15	< 0.001	0.27	< 0.001	0.36	< 0.001	0.43	< 0.001	0.46	< 0.001
50%	0.19	< 0.001	0.34	< 0.001	0.44	< 0.001	0.51	< 0.001	0.54	< 0.001
60%	0.23	< 0.001	0.40	< 0.001	0.50	< 0.001	0.57	< 0.001	0.59	< 0.001
70%	0.26	< 0.001	0.46	< 0.001	0.56	< 0.001	0.62	< 0.001	0.63	< 0.001
80%	0.30	< 0.001	0.51	< 0.001	0.61	< 0.001	0.66	< 0.001	0.66	< 0.001
90%	0.33	< 0.001	0.54	< 0.001	0.64	< 0.001	0.68	< 0.001	0.68	< 0.001

The “/” means that the independent variable was set as the reference (REF), therefore, there was no need to provide the *P* value

^a^*MDA* Methods of drug administration; *CDA* Coverage rate of drug administration

^b^Effects of different survey spots adjusted

**Table 3:** Analysis of CER on increased numbers of hygiene behaviors and increase on ratio of qualified hygiene behaviors under different coverage rate of health education by generalized linear models based on data from model simulation

Dependent variables	Coverage rate of health education (%)	1 year		2 years		3 years		4 years		5 years	
		Adjusted coefficient^a^	*P* value	Adjusted coefficient^a^	*P* value	Adjusted coefficient^a^	*P* value	Adjusted coefficient^a^	*P* value	Adjusted coefficient^a^	*P* value
CER on increased numbers of hygiene behaviors	10	1 (REF)	/	1 (REF)	/	1 (REF)	/	1 (REF)	/	1 (REF)	/
	20	− 102.72	< 0.01	− 76.71	< 0.01	− 68.81	< 0.01	− 66.15	< 0.01	− 68.02	< 0.01
	30	− 138.82	< 0.01	− 105.67	< 0.01	− 95.53	< 0.01	− 92.10	< 0.01	− 93.23	< 0.01
	40	− 158.17	< 0.01	− 122.26	< 0.01	− 110.97	< 0.01	− 106.58	< 0.01	− 107.94	< 0.01
	50	− 170.76	< 0.01	− 133.64	< 0.01	− 121.24	< 0.01	− 116.53	< 0.01	− 117.89	< 0.01
	60	− 179.91	< 0.01	− 142.21	< 0.01	− 128.70	< 0.01	− 123.30	< 0.01	− 123.79	< 0.01
	70	− 187.06	< 0.01	− 149.06	< 0.01	− 134.58	< 0.01	− 128.42	< 0.01	− 126.63	< 0.01
	80	− 192.92	< 0.01	− 154.74	< 0.01	− 139.05	< 0.01	− 131.40	< 0.01	− 127.32	< 0.01
	90	− 197.90	< 0.01	− 159.57	< 0.01	− 141.92	< 0.01	− 132.74	< 0.01	− 127.43	< 0.01
Increase on ratio of qualified hygiene behaviors	10	1 (REF)	/	1 (REF)	/	1 (REF)	/	1 (REF)	/	1 (REF)	/
	20	0.11	> 0.05	0.33	> 0.05	0.66	> 0.05	0.97	> 0.05	1.35	> 0.05
	30	0.22	> 0.05	0.70	> 0.05	1.48	> 0.05	2.23	> 0.05	3.13	> 0.05
	40	0.34	> 0.05	1.13	> 0.05	2.49	> 0.05	3.74	> 0.05	5.45	> 0.05
	50	0.46	< 0.05	1.61	> 0.05	3.68	> 0.05	5.59	> 0.05	8.41	> 0.05
	60	0.59	< 0.01	2.16	< 0.05	5.07	> 0.05	7.69	< 0.05	11.54	< 0.05
	70	0.72	< 0.01	2.76	< 0.01	6.69	< 0.05	10.14	< 0.01	14.24	< 0.01
	80	0.86	< 0.01	3.44	< 0.01	8.50	< 0.01	12.55	< 0.01	16.66	< 0.01
	90	1.01	< 0.01	4.18	< 0.01	10.12	< 0.01	14.60	< 0.01	18.92	< 0.01

The “/” means that the independent variable was set as the reference (REF), therefore, there was no need to provide the *P* value

*CER* cost-effectiveness ratio

^a^Effects of different survey spots adjusted

The model incorporated four distinct methods of drug administration, including WPD which involves mass drug administration to the entire population in designated areas; KPD targeting individuals aged 45 years and older for mass drug administration; ED where a certain proportion of residents undergo stool examination and positive cases receive free treatment; and EVD, where residents undergo stool examination and are encouraged to seek drug treatment voluntarily upon receiving positive results.

The cost-effectiveness of WPD and KPD was superior to that of EVD, and the higher the coverage of drug administration, the better the cost-effectiveness is for WPD and KPD. Yet the cost-effectiveness of ED and EVD was better in relatively low coverage. When all four methods of drug administration were incorporated into the model, the CER of WPD and KPD were comparatively lower than that of EVD. This implies that the cost needed to reduce one infected individual was lower, resulting in cost savings ranging from 384.79 to 504.64 CNY for WPD and 354.35 to 506.21 CNY for KPD per reduction in the number of infected persons (*P* < 0.001). Subsequently, when only WPD or KPD were considered in the model, the CER exhibited a gradual decrease as the coverage of drug administration increased overall. Across intervention durations of 1–5 years, the CER was found to be lowest at coverage rates of 80%, 60%, 50%, 40%, and 40% respectively. In comparison to a coverage rate of 10%, the cost required to reduce one infected person ranged from 49.53 to 122.53 CNY (*P* < 0.001). In light of ED and EVD, when the intervention measures are implemented for one year, the cost-effectiveness is more favorable at coverage rates of 20%, 30%, and 40% as opposed to 10%, resulting in cost savings of 41.23, 45.34, and 40.25 CNY per reduction in infected person. For interventions lasting 2–5 years, the CER was observed to be highest at a coverage rate of 90%, entailing an additional cost of 58.69, 149.68, 287.27, and 418.32 CNY per reduction in infected person (Table 1).

In general, WPD, ED, and EVD were superior to that of KPD in effect of hookworm disease control. When compared to KPD, WPD, ED, and EVD exhibited relatively higher reduction ratios in infection rates, ranging from 0.14 to 0.25, 0.10 to 0.19, and 0.08 to 0.17 (*P* < 0.001), respectively, over 1–5 years of intervention. In general, in comparison to a coverage rate of 10%, the CER decreases with increasing coverage rates, reaching its lowest point at a coverage rate of 90%. The reduction ratio in infection rates also increases with higher coverage rates, peaking at a coverage rate of 90% (Table 2).

For health education, the coverage rate increment means worse CER yet better effect. In the scenario of continuous health education spanning 1–5 years, in comparison to a reference coverage rate of 10%, the CER of health education diminishes gradually as coverage rates increase. Concurrently, the increment in the qualified rate of hygiene behavior rises steadily, indicating improved cost-effectiveness and intervention efficacy. At a health education coverage rate of 90%, the average cost savings per additional qualified individual in hygiene behavior ranges from 127.43 to 197.90 CNY as compared to the 10% coverage rate, with corresponding increases in the qualified rate of hygiene behavior ranging from 1.01 to 18.92 (Table 3).

## Discussion

Currently, there is limited research available on the cost-effectiveness analysis of hookworm disease [70, 71]. When addressing hookworm control, it is essential to consider both cost-effectiveness and efficacy, taking into account both short-term and long-term effects in terms of effectiveness. The cost-effectiveness model of hookworm disease in this study is trying to address the problem.

Researches have demonstrated the effectiveness of mass drug administration (MDA) in controlling hookworm infections, as evidenced by studies conducted in northern Ghana [32] and Sierra Leone [33]. The positive outcomes of ED have also been validated [37], aligning with the conclusions of this study. Moreover, based on the findings of the model simulation, WPD emerges as the most effective and cost-effective approach. However, in alignment with the technical program for the control of STHs, WPD is specifically recommended for areas with an infection rate of 20% or higher, taking ethical considerations into account. Integrating the simulation results with the control program guidelines, it is advised that in regions where the infection rate is ≥ 20%, WPD should be implemented alongside efforts to enhance the coverage rate of drug administration to achieve optimal effectiveness and cost-effectiveness. In areas where the infection rate falls below 20% but exceeds 5%, the inclusion of the other three drug administration methods in the selection list is recommended. In settings where adequate funding is available and the primary goal is to reduce the infection rate, ED could be considered. Conversely, in situations where funding is limited and a more favorable cost-effectiveness ratio is desired, KPD could be a more suitable option. It is recommended to enhance the coverage of these methods to the greatest extent possible, given the prevailing conditions. Mass drug administration in areas with an infection rate below 5% may have limited cost-effectiveness. Leveraging the existing national surveillance system to facilitate voluntary diagnosis and treatment of hookworm disease among local residents could enhance control efficiency.

This study has demonstrated that efforts should be directed towards achieving a drug administration coverage rate of 90% to enhance cost-effectiveness, where feasible. Other modeling studies also suggest that low-frequency, high-coverage deworming is more effective. For at a given coverage level, the lifespan of parasites in the environment limits the effectiveness of increased treatment frequency [38]. The selection of drug administration methods and coverage rates should be determined comprehensively, taking into account the funding availability and anticipated duration of the intervention. For instance, in interventions targeting the entire population or key populations over a period of 1–5 years, optimal cost-effectiveness can be achieved with drug administration coverage rates of 80%, 60%, 50%, 40%, and 40% respectively. The cost-effectiveness of ED and EVD is maximized when the deworming coverage rate is set at 10%. In economically disadvantaged regions, KPD could be a preferable strategy. It is relatively easier to implement with relatively better cost-effectiveness when the coverage of drug administration is limited. Additionally, the coverage for drug administration can be adjusted annually based on available funding to achieve optimal cost-effectiveness.

While health education may have a limited impact on reducing infection rates, it can play a significant role in improving the adherence to hygienic practices among the population, which is crucial for long-term control of hookworm disease. Therefore, in the context of hookworm disease control, it is advisable to integrate drug administration with health education efforts to not only decrease infection rates but also enhance the adoption of proper hygiene behaviors. Optimal cost-effectiveness and intervention outcomes can be achieved when the coverage rate of health education reaches 90%. This aligns with the current control strategy in China, which emphasizes the use of health education as a guiding principle and prioritizes the control of infection sources.

Other study conducted in China indicates that KPD is more effective for the control of hookworm disease and other soil-transmitted helminthiases, compared to MDA [70, 72]. Yet in light of this study, when compared to KPD, WPD, ED, and EVD exhibited relatively higher reduction ratios in infection rates. The observed inferior intervention effect of KPD compared to ED and EVD can be attributed to specific factors. In this study, the key and occupational exposure population was defined as individuals aged 45 and above. When considering the same coverage rate of drug administration, the actual coverage rate for KPD is determined by the product of the proportion of key populations and the drug administration coverage rate for these populations, which tends to be lower than other methods. While KPD may yield better results in terms of infection rate among key populations, as this group typically exhibits higher infection rates than the general population, it is essential to prioritize targeting all susceptible individuals rather than focusing on specific subgroups. This is crucial for achieving the overarching goal of effectively controlling and eliminating hookworm disease. Moreover, other studies have shown that targeted deworming programs for children have limited effectiveness in controlling hookworm infections [35], as children are highly susceptible to reinfection in environments where both adults and children are infected [36]. Therefore, in China, the key demographic for control of hookworm disease should be middle aged and older people who exhibit higher infection rates, rather than children.

There are certain limitations in this study. The model is based on certain assumptions and, due to practical limitations, does not encompass all factors related to the endemicity and control of hookworm disease. It only includes only key factors that were identified through expert consultation, potentially impacting its applicability. Also, during the modeling process, data unavailable from field surveys were sourced from yearbooks or literature. Historical temperature, humidity, vegetation index, etc., at local spots were estimated using spatial interpolation. These data limitations impact the accuracy of the model. Moreover, the complexity of the model's structure also poses a risk of overfitting. Finally, during the survey period, the COVID-19 pandemic was widespread, leading to reduced outdoor activities among residents and, consequently, a lower risk of hookworm infection. However, this also limited opportunities for seeking medical attention, which potentially affected the accuracy of survey results.

The field investigation conducted over a 3-year period. However, to further enhance the applicability and longevity of the model, long-term continuous intervention and field investigations are essential to validate the model over extended timeframes. This ongoing validation process will enable the expansion of the model's application duration. Additionally, the model can be continuously enriched by incorporating new feasible control measures, such as the potential inclusion of vaccine [73]. Furthermore, while the model was developed based on the specific context of China, it can be adapted and tailored to accommodate the unique circumstances of other regions worldwide, thereby serving as a valuable tool to guide global efforts in the control of hookworm disease. Lastly, while the current model primarily focuses on the transmission dynamics of *N. americanus*, it can be modified to simulate the transmission and control strategies for *A. duodenale* and other parasitic diseases.

## Conclusions

This study presents optimized control strategies that emphasize both cost-effectiveness and control outcomes for the hookworm disease control program in China. Future control efforts should tailor measures to the endemic status and local conditions of different areas to reduce the infection rate of hookworm disease, ultimately aiming to control and eliminate hookworm disease in China. Moreover, this study also highlights the important implications of implementing drug administration measures according to endemic status for hookworm diseases control in other counties.

## Supplementary Information

Supplementary Material 1: Fig. S1. Diagram of the research project for constructing an interventional mechanism model of hookworm disease. Fig. S2. System framework diagram for interventional mechanism model of hookworm disease. Fig. S3. Importance of indicators in interventional mechanism model of hookworm disease obtained through Delphi method. Fig. S4. Results of integral error test for interventional mechanism model of hookworm disease. Fig. S5. Results of sensitivity test for interventional mechanism model of hookworm disease. Fig. S6. Results of model simulation based on field data for interventional mechanism model of hookworm disease

## Data Availability

Following approval by the National Institute of Parasitic Diseases, Chinese Center for Disease Control and Prevention (Shanghai, China), the datasets underlying the results of this article will be made available to investigators. Please email the corresponding author for more information.

## Abbreviations

CER: Cost effectiveness ratio
CLD: Causal loop diagram
ED: Examination and deworming
EVD: Examination and voluntarily deworming
KPD: Key population deworming
MDA: Mass drug administration
NDVI: Normalized difference vegetation index
NTD: Neglected tropical disease
SDGs: Sustainable Development Goals
SFC: Stock flow chart
STH: Soil-transmitted helminth
STHs: Soil-transmitted helminthiases
WASH: Water, sanitation, and hygiene
WHO: World Health Organization
WPD: Whole population deworming
