# Supplementary material for: Optimal control strategies supported by system dynamics modelling: a study on hookworm disease in China
Source: Infect Dis Poverty. 2025 Mar 19;14:22. doi: 10.1186/s40249-025-01293-w (PMC11921666; doi:10.1186/s40249-025-01293-w)
Supplement: Supplementary file 1 — Supplementary Material 1: Fig. S1. Diagram of the research project for constructing an interventional mechanism model of hookworm disease. Fig. S2. System framework diagram for interventional mechanism model of hookworm disease. Fig. S3. Importance of indicators in interventional mechanism model of hookworm disease obtained through Delphi method. Fig. S4. Results of integral error test for interventional mechanism model of hookworm disease. Fig. S5. Results of sensitivity test for interventional mechanism model of hookworm disease. Fig. S6. Results of model simulation based on field data for interventional mechanism model of hookworm disease [file 40249_2025_1293_MOESM1_ESM.docx]

**Supplementary materials includes:**

Figs. S1 to S6

Tables S1 to S7

Fig. S1.

Diagram of the research project for constructing an interventional mechanism model of Hookworm disease.

Fig. S2.

System framework diagram for interventional mechanism model of Hookworm disease.

Fig. S3.

Importance of indicators in interventional mechanism model of Hookworm disease obtained through Delphi method.


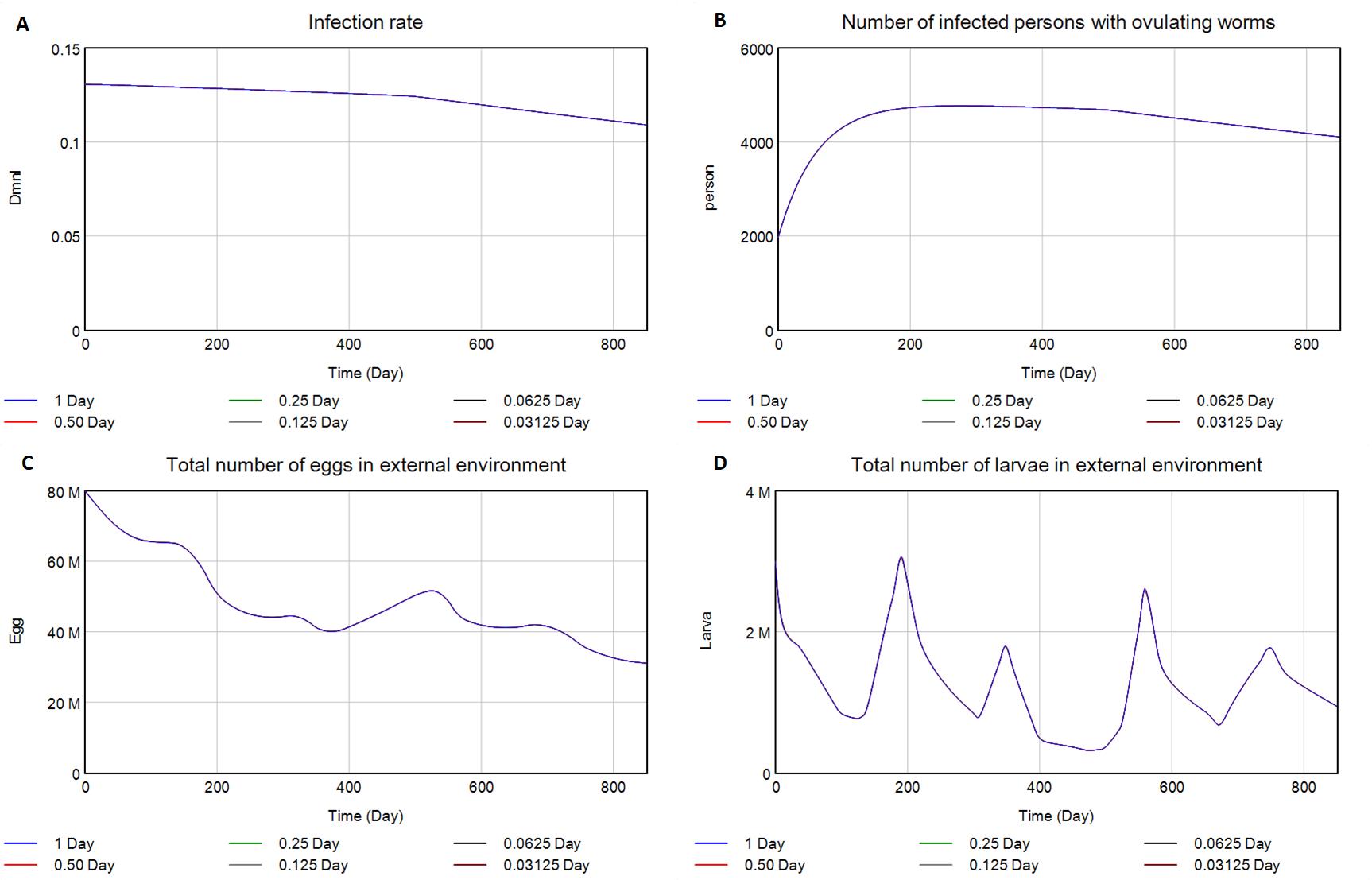


Fig. S4.

Results of integral error test for interventional mechanism model of Hookworm disease.


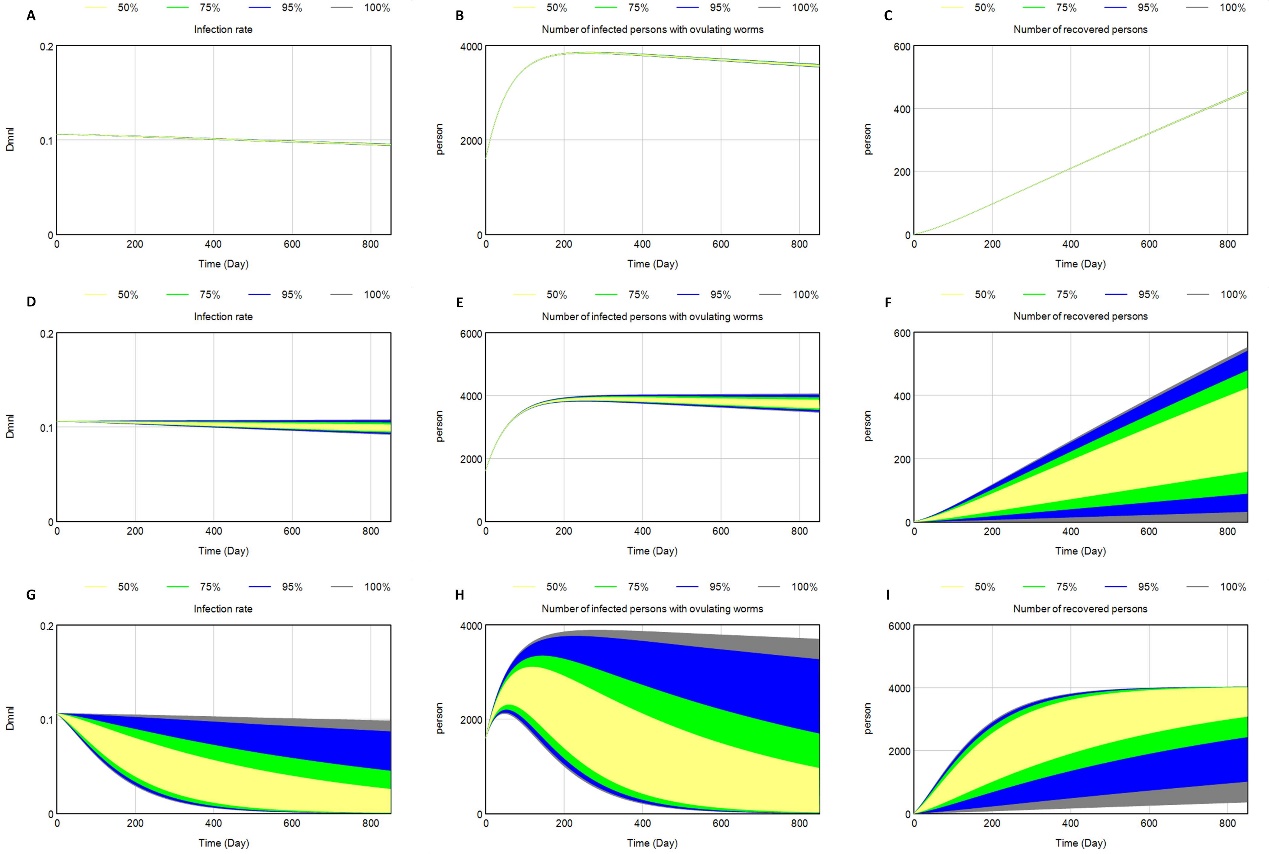


Fig. S5.

Results of sensitivity test for interventional mechanism model of Hookworm disease.


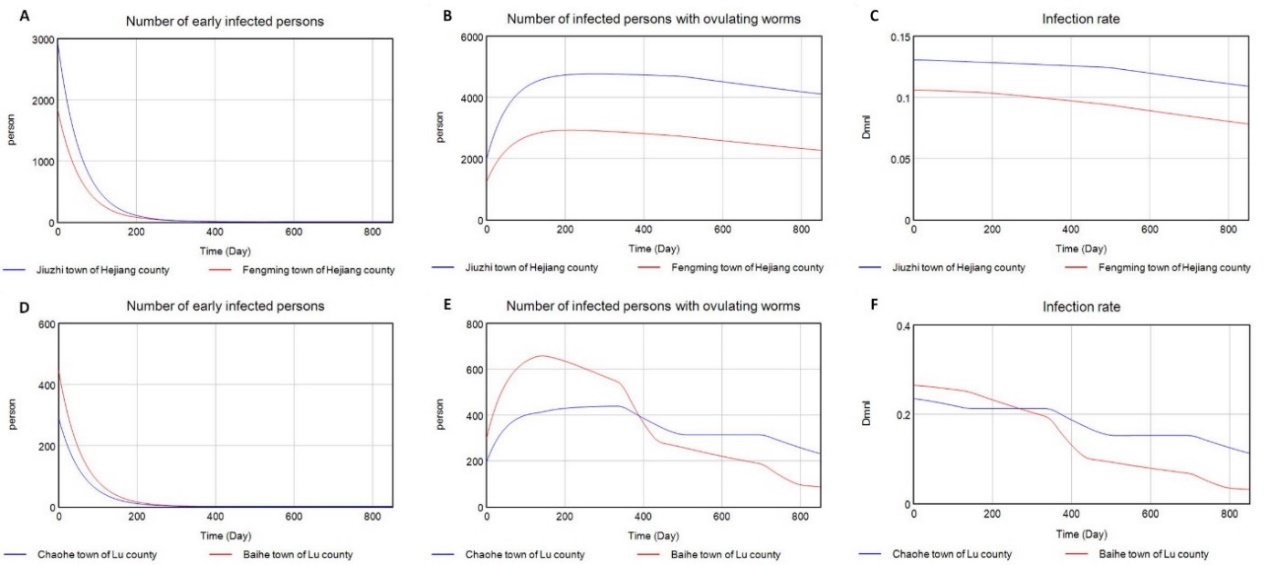


Fig. S6.

Results of model simulation based on field data for interventional mechanism model of Hookworm disease.

Table S1.

State variables in the interventional mechanism model of Hookworm disease.

| **Equations of level variables** | **Units** |
| --- | --- |
| Number of susceptibles=INTEG(-Rate of infection, Number of permanent residents*(1-Infection rate)) | person |
| Number of early infected persons=INTEG(Rate of infection-Maturation rate of worms 1, Initial number of infected people*0.6) | person |
| Number of infected persons with ovulating worms=INTEG(Maturation rate of worms 1-Proportion rate of recovery 2, Initial number of infected people*0.4) | person |
| Number of recovered persons= INTEG (Proportion rate of recovery 2+Proportion rate of recovery 3+Proportion rate of recovery 4-Rate of recovery, 0) | person |
| Number of re-infected persons=INTEG(Rate of recovery-Proportion rate of recovery 3-Maturation rate of worms 2, 0) | person |
| Number of re-infected persons with ovulating worms=INTEG(Maturation rate of worms 2-Proportion rate of recovery 4, 0) | person |
| Total number of eggs in external environment=INTEG(Rate of ovulation-Development rate of eggs, 8e+07) | egg |
| Total number of larvae in external environment=INTEG(Development rate of eggs-Death rate of larva, 3e+06) | larva |

Table S2.

Rate variables in the interventional mechanism model of Hookworm disease.

| **Equations of rate variables** | **Units** |
| --- | --- |
| Rate of infection=Number of susceptibles*Probability of infection for each susceptible*IF THEN ELSE(Number of susceptibles>0, 1 , 0 ) | person/day |
| Maturation rate of worms 1= Number of early infected people/Maturation time length of worms*IF THEN ELSE(Number of early infected people>=1, 1 , 0 ) | person/day |
| Maturation rate of worms 2=Number of re-infected person/Maturation time length of worms*IF THEN ELSE(Number of re-infected person >=1, 1 , 0 ) | person/day |
| Rate of reinfection=Number of recovered person*Probability of infection for each susceptible*IF THEN ELSE (Number of recovered person>=1, 1, 0 ) | person/day |
| Recovery rate 1= (Number of infected persons with ovulating worms*Proportion of recovery 2)*IF THEN ELSE(Number of infected person with ovulating worms>=1, 1 , 0 ) | person/day |
| Recovery rate 2=Number of re-infected person*Proportion of recovery 1*IF THEN ELSE(Number of re-infected person>=1, 1, 0 ) | person/day |
| Recovery rate 3= (Number of re-infected person with ovulating worms*Proportion of recovery 2)*IF THEN ELSE(Number of re-infected person with ovulating worms>=1, 1 , 0) | person/day |
| Rate of ovulation=Number of female worms that ovulating in infected persons*Average number of ovulation per female worm per day | egg/day |
| Development rate of eggs=Total number of eggs in external environment*Proportion of eggs reaching fields *Hatching ratio of eggs/4.5*IF THEN ELSE(Total number of eggs in external environment >0, 1 , 0 ) | egg/day |
| Death rate of larva=Total number of larvae in external environment/6.5*IF THEN ELSE(Total number of larvae in external environment>0, 1, 0 ) | larva/day |

Table S3.

Other auxiliary variables in the interventional mechanism model of Hookworm disease.

| **Equations of other auxiliary variables*** | **Units** |
| --- | --- |
| ADNPAFMF=RDNPAFMF-((NPR-INPAFMF)*CRHE*ERHEFMF*IF THEN ELSE(RDNPAFMF-((NPR-INPAFMF)*CRHE*ERHEFMF)>0, 1 , 0 )) | Person |
| ADNPCSBBW=RDNPCSBB-(CRHE*ERHEPCSBB *(NPR-INPCSBB)*IF THEN ELSE ( RDNPCSBB-CRHE*ERHEPCSBB*(NPR-INPCSBB)>0 , 1 , 0)) | Person |
| AINPUSL=RINPUSL-(INPUSL*CRHE*ERHEPUSL *IF THEN ELSE(RINPUSL-(INPUSL*CRHE *ERHEPUSL)>0, 1 , 0 )) | Person |
| AINPVSD=RINPVSD-(INPVADT*CRHE*ERHEVDT)*IF THEN ELSE(RINPVSD -(INPVADT*CRHE*ERHEVDT)>0, 1 , 0) | Person |
| ANAWIP=NFWOIP/(NIPOW +NRPOW)*2 | Worm |
| ANLCP=TNLEN/TAA*(PRP*NPR/TAA ) | Larva |
| ANOFWD=7500*EXP(-0.05*ANAWIP) | Egg |
| CRSBBW= (INPCSBB-ADNPCSBBW)/NPR*IF THEN ELSE((INPCSBB-ADNPCSBBW)>=0 ,1, 0 ) | Dmnl |
| HRE=HRE 1*HRE 2*HRE 3 *HRE 4*(1-PLLOR) | Dmnl |
| HRE 1= WITH LOOKUP (TEMPERATURE, ([(0,0)-(10,10)],(-30,0),(-5,0.01),(0,0.1),(5,0.2),(10,0.5),(15,0.6),(20,0.7),(25,0.8),(30,0.9),(35,0.8),(40,0.1),(45,0), (50,0) )) | Dmnl |
| HRE 2=WITH LOOKUP (HUMIDITY, ([(0,0)-(10,10)],(0,0),(10,0.1),(20,0.2),(30,0.2),(35,0.3),(40,0.3),(45,0.4),(50,0.4),(55,0.5),(60,0.5), (70,0.6),(80,0.7),(90,0.9),(100,0.6) )) | Dmnl |
| HRE 3= WITH LOOKUP (ITL, ([(0,0)-(10,10)],(0,0),(20,0.2),(30,0.3),(40,0.4),(50,0.5),(60,0.6),(70,0.7),(80,0.8),(85,0.9),(90,0.9),(95,0.8),(100,0.7),(150,0.2) ,(200,0.1))) | Dmnl |
| HRE 4= WITH LOOKUP (NDVI, ([(0,0)-(10,10)],(0,0),(0.1,0.5),(0.2,0.6),(0.3,0.8),(0.4,0.9),(0.5,0.9), (0.6,0.9),(0.7,0.9),(0.8,0.9),(0.9,0.6), (1,0.3) )) | Dmnl |
| IRKP=RIRKP*TIR | Dmnl |
| IANFWEIP=IAII/120 | Worm |
| INIP=NPR*IIR | Person |
| INPAFMF=IRFMF*NPR | Person |
| INPCSBB=ICRSBBW*NPR | Person |
| INPUSL= IRPUSL*NPR | Person |
| INPVADT=IRPVADT*NPR | Person |
| NEPADTF=NEPIH*PEPADT | Person |
| NEPIH=NPBE*TIR*SE | Person |
| NFWOIP=INTEGER( DELAY1(((NIPOW+ NRPOW)*IANFWEIP*BRR),70))/TLHLHB | Worm |
| NIWPAFDT=CRDTWP*(TNI-(NEPADTF+NPVADT))*IF THEN ELSE( (TNI-(NEPADTF +NPVADT))>0 , 1 ,0 ) | Person |
| NKPIHAFDT = ((NPR*PKP*PKPAFDT*IRKP)-(NPVADT+NEPADTF)*PKP*PKPAFDT)*IF THEN ELSE (((NPR*PKP*PKPAFDT *IRKP)-(NPVADT +NEPADTF)*PKP *PKPAFDT)>=0, 1 , 0 ) | Person |
| NPAHE=CRHE*NPR | Person |
| NPBE=NPR*PPBE | Person |
| NPVADT=NEPIH*(1-PEPADT)*PPVADT | Person |
| PCLP=1-EXP(-(ANLCP)) | Dmnl |
| PCLS=((PCLP*(NS/NPR )*CRSBBW*PKP *2.23)+(PCLP*(NS/ NPR)*CRSBBW *(1-PKP)*0.51)) | Dmnl |
| PIES=PIESCL *PCLS | Dmnl |
| PIESCL=1-(EXP(-PIESCOL)) | Dmnl |
| PERF= (1-RSLU*PSL)*RFMF *(1-PELOR) | Dmnl |
| PPVADT= (INPVADT+ AINPVSD)/NPR*IF THEN ELSE( (INPVADT+ AINPVSD)<=NPR, 1 , NPR/ (INPVADT+ AINPVSD)) | Dmnl |
| PR 1=TCRDT*DE/RT 1*TDT | Dmnl |
| PR 2=TCRDT*DE/RT 2*TDT | Dmnl |
| RFMF=(INPAFMF-ADNPAFMF)/NPR*IF THEN ELSE( (INPAFMF-ADNPAFMF)>=0, 1 , 0) | Dmnl |
| RSLU= (INPUSL+AINPUSL)/NPR*IF THEN ELSE( (INPUSL +AINPUSL)<=NPR, 1 , NPR/(INPUSL+AINPUSL)) | Dmnl |
| RDNPAFMF=CRHE*NPR*((1+ERHEFMF )^INTEGER(TIME/365+0.999)-1)*IF THEN ELSE(((1+ERHEFMF )^INTEGER(TIME/365+0.999)-1)<=1, 1,1/((1+ERHEFMF )^INTEGER(TIME/365+0.999)-1) ) | Person |
| RDNPCSBB =NPAHE*((1+ERHEPCSBB )^INTEGER(TIME/365+0.999)-1)*IF THEN ELSE( ((1+ERHEPCSBB )^INTEGER(TIME/365+0.999)-1) <=1, 1 , 1/((1+ERHEPCSBB )^INTEGER(TIME/365+0.999)-1)) | Person |
| RINPUSL=CRHE*NPR*((1+ERHEPUSL )^INTEGER(TIME/365+0.999)-1)*IF THEN ELSE( ((1+ERHEPUSL)^INTEGER(TIME/365+0.999)-1)<=1 , 1 , 1/((1+ERHEPUSL )^INTEGER(TIME/365+0.999)-1) ) | Person |
| RINPVSD=CRHE*NPR*((1+ERHEVDT)^INTEGER(TIME/365+0.999)-1)*IF THEN ELSE(((1+ERHEVDT )^INTEGER(TIME/365+0.999)-1)<=1 , 1 , 1/((1+ERHEVDT)^INTEGER(TIME/365+0.999)-1) ) | Person |
| TCRDT=TNIPADT/TNI | Dmnl |
| TIR=TNI/NPR | Dmnl |
| TNIPADT=NIWPAFDT+NEPADTF +NPVADT+NKPIHAFDT | Person |
| TNI=NEIP+NRP+NRPOW +NIPOW | Person |

*Full names of variable Abbreviations in Table S3:

ADNPAFMF: Absolute decreased number of people applying fresh manure fertilization

ADNPCSBBW: Absolute decreased number of people contact with soil in barehanded or barefoot way

AINPUSL: Absolute increased number of people using sanitary latrines

AINPVSD: Absolute increased number of people voluntarily seeking for drugs

ANAWIP: Average number of adult worms per infected person

ANLCP: Average number of larva contacting per person

ANOFWD: Average number of ovulation per female worm per day

BRR: Basic reproduction rate

CRDTWP: Coverage rate of drug treatment for whole population

CRHE: Coverage rate of health education

CRSBBW: Contact rate with soil in barehanded or barefoot way

DE: Drug efficiency

ERHEFMF: Effective rate of health education on fresh manure fertilization

ERHEPCSBB: Effective rate of health education on people contacting soil with barehand or barefoot

ERHEPUSL: Effective rate of health education on people using sanitary latrines

ERHEVDT: Effective rate of health education on voluntary drug treatment

HRE: Hatching ratio of eggs

IAII: Initial average infection intensity

IANFWEIP: Initial average number of female worms in each infected person

ICRSBBW: Initial contact rate with soil in barehanded or barefoot way

IIR: Initial infection rate

INIP: Initial number of infected people

INPAFMF: Initial number of people applying fresh manure fertilization

INPCSBB: Initial number of people contacting soil with barehand or barefoot

INPUSL: Initial number of people using sanitary latrines

INPVADT: Initial number of people voluntarily accepting drug treatment

IRFMF: Initial rate of fresh manure fertilization

IRKP: Infection rate of key population

IRPUSL: Initial rate of people using sanitary latrines

IRPVADT: Initial rate of people voluntarily accepting drug treatment

ITL: Illumination time length

NDVI: Normalized difference vegetation index

NEIP: Number of early infected people

NEPADTF: Number of examined people accepting drug treatment for free

NEPIH: Number of examined people infected with hookworm

NFWOIP: Number of female worms that ovulating in infected persons

NIPOW: Number of infected persons with ovulating worms

NIWPAFDT: Number of infected whole population accepting free drug treatment

NKPIHAFDT: Number of key population infected with hookworm accepting free drug treatment

NPAHE: Number of people accepting health education

NPBE: Number of people being examined

NPR: Number of permanent residents

NPVADT: Number of people voluntarily accepting drug treatment

NRP: Number of re-infected person

NRPOW: Number of re-infected persons with ovulating worms

NS: Number of susceptibles

PCLP: Probability of contacting larva per person

PCLS: Probability of contacting larva per susceptible

PELOR: Proportion of eggs loss due to other reasons

PEPADT: Proportion of examined people accepting drug treatment

PERF: Proportion of eggs reaching fields

PIES: Probability of infection for each susceptible

PIESCL: Probability of infection for each susceptible contacting larva

PIESCOL: Probability of infection for each susceptible contacting one larva

PKP: Proportion of key population

PKPAFDT: Proportion of key population accepting free drug treatment

PLLOR: Proportion of larva loss due to other reasons

PPBE: Proportion of people being examined

PPVADT: Proportion of people voluntarily accepting drug treatment

PR: Proportion of recovery

PRP: Proportion of rural population

PSL: Proportion of sanitary latrines

RDNPAFMF: Relatively decreased number of people applying fresh manure fertilization

RDNPCSBB: Relatively decreased number of people contacting soil with barehand or barefoot

RFMF: Rate of fresh manure fertilization

RINPUSL: Relatively increased number of people using sanitary latrines

RINPVSD: Relatively increased number of people voluntarily seeking for drugs

RIRKP: Ratio of infection rate in key population

RSLU: Rate of sanitary latrines usage

RT: Recovery time

SE: Sensitivity of examination

TAA: Total areas for agriculture

TCRDT: Total coverage rate of drug treatment

TDT: Times of drug treatment

TIR: Total infection rate

TLHLHB: Time length of hookworm living in human body

TNI: Total number of infection

TNIPADT: Total number of infected people accepting drug treatment

TNLEN: Total number of larvae in external environment

Table S4.

Constant and exogenous variables in the interventional mechanism model of Hookworm disease.

| **Constant and exogenous variables** | **Units** |
| --- | --- |
| Basic reproduction rate | dmnl |
| Coverage rate of drug treatment for whole population | dmnl |
| Coverage rate of health education | dmnl |
| Drug efficiency | dmnl |
| Effective rate of health education on fresh manure fertilization | dmnl |
| Effective rate of health education on people contacting soil with barehand or barefoot | dmnl |
| Effective rate of health education on people using sanitary latrines | dmnl |
| Effective rate of health education on voluntary drug treatment | dmnl |
| Relative humidity | dmnl |
| Illumination time length | day |
| Initial average infection intensity | gram/egg |
| Initial contact rate with soil in barehanded or barefoot way | dmnl |
| Initial infection rate | dmnl |
| Initial number of permanent residents | person |
| Initial rate of fresh manure fertilization | dmnl |
| Initial rate of people using sanitary latrines | dmnl |
| Initial rate of people voluntarily accepting drug treatment | dmnl |
| Maturation time length of worms | day |
| Natural population growth rate | dmnl |
| Normalized Difference Vegetation Index | dmnl |
| Number of permanent residents | person |
| Probability of infection for each susceptible contacting one larva | dmnl |
| Proportion of examined people accepting drug treatment | dmnl |
| Proportion of eggs loss due to other reasons | dmnl |
| Proportion of key population | dmnl |
| Proportion of key population accepting free drug treatment | dmnl |
| Proportion of larva loss due to other reasons | dmnl |
| Proportion of people being examined | dmnl |
| Proportion of rural population | dmnl |
| Proportion of sanitary latrines | dmnl |
| Ratio of infection rate in key population | dmnl |
| Recovery time 1 | day |
| Recovery time 2 | day |
| Sensitivity of examination | dmnl |
| Temperature | ℃ |
| Time length of hookworm living in human body | day |
| Times of drug treatment | time |
| Total areas for agriculture | square meter |

Table S5.

Relative errors of indicators for hygiene habits according to model simulation.

| Survey spots | Indicators of hygiene habits | Initial value (%) | Actual value (%) | Prediction value (%) | Relative error (%) |
| --- | --- | --- | --- | --- | --- |
| Fengming town | Rate of sanitary latrines usage | 0.50 | 0.78 | 0.81 | 3.62 |
|  | Contact rate with soil in barehanded or barefoot way | 0.93 | 0.79 | 0.70 | 10.89 |
|  | Rate of fresh manure fertilization | 0.60 | 0.35 | 0.38 | 9.71 |
|  | Proportion of people voluntarily seeking for drugs | 0.81 | 0.85 | 0.86 | 1.65 |
| Baihe town | Rate of sanitary latrines usage | 0.65 | 0.78 | 0.80 | 3.11 |
|  | Contact rate with soil in barehanded or barefoot way | 0.81 | 0.66 | 0.63 | 4.69 |
|  | Rate of fresh manure fertilization | 0.22 | 0.22 | 0.19 | 12.02 |
|  | Proportion of people voluntarily seeking for drugs | 0.97 | 0.95 | 0.98 | 2.88 |

Table S6.

Relative errors of infection rates according to model simulation.

| Survey spots | Initial value (%) | Actual value (%) | Prediction value (%) | Relative error (%) |
| --- | --- | --- | --- | --- |
| Fengming town | 10.62 | 7.2 | 8.27 | 14.86 |
| Jiuzhi town | 13.06 | 12.2 | 12.6 | 3.28 |
| Baihe town | 26.5 | 3.08 | 3.5 | 13.64 |
| Chaohe town | 23.5 | 13.54 | 12.6 | 6.94 |

Table S7.

Equations of variables for cost-effectiveness model of hookworm disease.

| **Equations of Variables*** | **Units** |
| --- | --- |
| TCDC= INTEG (IRC, 0) | Yuan |
| TCHE= INTEG (IRCHE, 0) | Yuan |
| IRC=ACWPDA+ACDDA+ACHE+ACKPDA+IF THEN ELSE( (ACWPDA+ACDDA+ACHE+ACKPDA) >0, IC, 0 ) | Yuan/Year |
| IRCHE=ACHE+ IF THEN ELSE (ACHE >0, IC ,0 ) | Yuan/Year |
| ACDDA=NPBE*TIR*PCDA*PEPADA+NPBE*PCD | Yuan |
| ACHE=NPAHE*PCHE | Yuan |
| ACKPDA=NKPRDA*PCDA | Yuan |
| ACWPDA=NWPADA*PCDA | Yuan |
| CDNIP=CDIR/NPR | Yuan/Person |
| CDIR=TCDC/IF THEN ELSE( DIR >0 , DIR , TCDC+1 )*IF THEN ELSE (IT=14, 1 , 0 ) | Yuan |
| CINHB=CIQRHB/NPR | Yuan/Person |
| CIQRHB=TCHE/IF THEN ELSE( IRQHB >0, IRQHB, 1E+12 ) | Yuan/Person |
| DIR=IIR-TIR | Dmnl |
| IRQHB=QRHBQRHB | Dmnl |
| QRHB= (1-RFMF)*RSLU*PPVADA*(1-CRSBBW) | Yuan |

*Full names of variable Abbreviations in Table S7:

ACDDA: Annual cost of diagnosis and drug administration

ACHE: Annual cost of health education

ACKPDA: Annual cost of key population drug administration

ACWPDA: Annual cost of whole population drug administration

CDIR: CER on decreasement of infection rate

CDNIP: CER on decreased number of infected people

CINHB: CER on increasement numbers of hygiene behaviors

CIQRHB: CER on increasement of qualified rate of hygiene behaviors

CRSBBW: Contact rate with soil in barehanded or barefoot way

DIR: Decreasement of infection rate

IC: Initiating cost

IIR: Initial infection rate

IRC: Increase rate of cost

IRCHE: Increase rate of cost for health education

IRQHB: Increasement on ratio of qualified hygiene behaviors

IT: Indicator timepoint

NKPRDA: Number of key population receiving drug administration

NPAHE: Number of population accepting health education

NPBE: Number of population being examined

NPR: Number of permanent residents

NWPADA: Number of whole population accepting drug administration

PCD: Per cost of diagnosis

PCDA: Per cost of drug administration

PCHE: Per cost of health education

PEPADA: Proportion of examined people accepting drug administration

PPVADA: Proportion of people voluntarily accepting drug administration

QRHB: Qualified rate of hygiene behaviors

QRHBQRHB: Qualified rate of hygiene behaviors-initial qualified rate of hygiene behaviors

RFMF: Rate of fresh manure fertilization

RSLU: Rate of sanitary latrines usage

TCDC: Total cost of disease control

TCHE: Total cost of health education

TIR: Total infection rate
